# Overview of systematic reviews and meta-analyses on problematic video game use and gaming disorder in adolescents and young adults

**DOI:** 10.1016/j.eclinm.2026.104195

**Published:** 2026-09-28

**Authors:** Jason M. Nagata, Christiane K. Helmer, Oliver H. Huang, Sahana Nayak, Kevin Bao, Stephanie Zhang, Kevin Handoko, Elizabeth J. Li, Megan A. Moreno, Jason M. Lavender

**Affiliations:** a Division of Adolescent and Young Adult Medicine, Department of Pediatrics, University of California, San Francisco, 550 16th Street, Box 0503, San Francisco, CA, 94143, USA; b Department of Pediatrics, University of Wisconsin, 500 Lincoln Dr, Madison, WI, USA; c Military Cardiovascular Outcomes Research Program (MiCOR), Department of Medicine, Uniformed Services University of the Health Sciences, 4301 Jones Bridge Rd, Bethesda, MD, 20814, USA; d The Metis Foundation, 84 NE Interstate 410 Loop # 325, San Antonio, TX, 78216, USA

**Keywords:** Video games, Gaming disorder, Internet gaming disorder, Problematic video game use, Adolescent, Youth

## Abstract

**Background:**

Problematic video game use and disordered gaming are increasingly recognized adolescent health concerns.

**Methods:**

This overview summarizes assessment, epidemiology, risk factors, health outcomes, and treatment of problematic and disordered gaming among adolescents and young adults. PubMed, Web of Science, and PsycInfo were searched for reviews published from 9/1/2016 to 5/26/2026. At least two reviewers screened and extracted data. Risk-of-bias assessments were summarized. This review is registered with the International Platform of Registered Systematic Review and Meta-analysis Protocols (INPLASY #202670067).

**Findings:**

Of 98 unique records, 51 were included. Prevalence varied by region, sociodemographics, and diagnostic definitions, with gaming disorder or internet gaming disorder affecting 6–10% of adolescents and young adults worldwide. Earlier gaming initiation, maladaptive gaming patterns, poorer mental health, and controlling parenting were associated with increased risk. Problematic gaming was linked to adverse physical, sleep, neurobiological, and mental health outcomes. Cognitive behavioral therapy showed the strongest therapeutic evidence. Evidence certainty was low-to-moderate.

**Interpretation:**

The findings highlight the importance of early identification of problematic and disordered gaming among youth who begin gaming young, or experience mental health symptoms, social difficulties, or limited parental support. Future research should refine diagnostic criteria, evaluate treatments through randomized trials, and clarify developmental mechanisms.

**Funding:**

JMN was supported by the National Institutes of Health and Rise Together, a donor-advised fund sponsored and administered by the National Philanthropic Trust.

## Introduction

Video games are now a central part of adolescent life: more than 90% of adolescents play video games.^1^ Contemporary video games encompass a wide array of potential benefits, including social connection, cognitive engagement, physical activity (e.g., exergaming), and educational enrichment.^2^ Yet, a subset of adolescents engage in gaming patterns that are maladaptive or impair functioning.^3^^,^^4^ Importantly, evidence suggests that potential harms are not uniform across all gaming, but depend heavily on specific content, contexts, and patterns of use.^5^ This more differentiated perspective reflects a broader shift in the digital health field, moving away from assumptions that all screen time or gaming is inherently harmful, and toward identifying precise mechanisms of risk, an approach highlighted in recent critiques of overly broad and monocausal technological explanations.^5^

A major challenge has been the lack of consensus on definitions and measurement.^3^^,^^4^ Terms such as problematic video game use have been operationalized in multiple ways, and although the World Health Organization's International Classification of Diseases 11th Revision (ICD-11)^6^ now recognizes gaming disorder, the criteria remain debated, and the corresponding Diagnostic and Statistical Manual of Mental Disorders, Fifth Edition (DSM-5) construct, internet gaming disorder, has not been fully adopted.^7^^,^^8^ This highlights the need for a careful and critical synthesis of what is known and where uncertainties remain.

The objective of this review is to synthesize the current state of evidence from systematic reviews and meta-analyses on problematic video game use and disordered gaming in adolescents and young adults. We examine the history of the construct, current definitions and assessment tools, epidemiology, risk factors, associated mental and physical health correlates, and emerging strategies for prevention and treatment. Throughout, we highlight areas of consensus and ongoing controversy, identify gaps in measurement and theory, and outline priorities for future research and clinical guidance for pediatricians.

Concerns about video game use, particularly in youth, were first described in 1983 by Soper's report “Junk-Time Junkies: An Emerging Addiction Among Students.”^7^ Soper highlighted various factors that could contribute to the development of gaming addiction in students (Panel 1). Research during the 1990s further investigated video game addiction, using the Diagnostic and Statistical Manual of Mental Disorders (DSM), Third and Fourth Edition pathological gambling criteria as a framework to assess problematic video gaming.^7^^,^^8^ The advent of online games in the 2000s led to expanded interest in studying problematic video game use, particularly with regard to online video gaming. In 2013, the American Psychiatric Association (APA) added internet gaming disorder as a disorder for future research in section III of the DSM-5.^9^ In 2019, gaming disorder was included in the World Health Organization's 11th revision of the International Classification of Diseases (ICD-11),^10^ though it did not become a fully ratified disorder until 2022.^6^^,^^11^ The ICD-11 also included the term hazardous gaming, which refers to a gaming pattern that may be harmful to the individual or others around them, but does not rise to the clinical diagnosis of gaming disorder.^6^ Notably, while internet gaming disorder and gaming disorder refer to clinical diagnoses, problematic video game use is a broader construct. It is sometimes used to refer solely to gaming that is harmful but subclinical. However, this term can also be a catchall for both clinical and nonclinical patterns of harmful gaming. In this review, we use disordered gaming as a term when referring to a clinical diagnosis, and problematic video game use (or problematic video gaming) as the broader term for excessive and/or maladaptive gaming behavior.

Panel 1 **History of internet gaming disorder and gaming disorder**

1983: The first paper on gaming disorder emerges in a school counseling journal entitled “Junk Time Junkies: An Emerging Addiction Among Students” by Soper and Miller.

1990s: Additional studies in the 1990s utilize the DSM-III and DSM-IV's definition of pathological gambling to assess problematic video game use.

2000s: Gaming disorder research begins to study online and multiplayer gaming.

2013: Internet gaming disorder is added to the DSM-5 in Section III as a condition for further study.

2019: Gaming disorder is added to the International Classification of Diseases 11th Revision.

The DSM-5 characterizes internet gaming disorder as persistent online gaming causing significant impairment or distress over a 12-month period, with five or more of nine criteria (e.g., preoccupation, withdrawal symptoms; see Table 1) required for a diagnosis.^9^ One major criticism of this polythetic approach is that it can lead to excessive diagnostic heterogeneity,^4^ such that two patients with the diagnosis could exhibit few overlapping symptoms. However, heterogeneity in symptom presentation is not unique to internet gaming disorder and is characteristic of most DSM-5 diagnoses. Many other diagnoses in the DSM-5 rely on threshold-based criteria composed of partially distinct symptoms. Additionally, some argue that certain criteria do not accurately distinguish between addicted and non-addicted disorders.^3^ For instance, gamers with and without gaming disorder endorsed playing video games to escape negative emotions.^12^ Importantly, beyond meeting individual criteria, a diagnosis requires significant distress and/or impairment. In contrast to the DSM-5, the ICD-11 classifies gaming disorder into two categories, predominantly online and predominantly offline, with the former involving persistent gaming conducted primarily over the internet and the latter involving persistent gaming that occurs largely without internet use.^6^ Both subtypes share three core features: impaired control over gaming, increasing prioritization of gaming over other life interests and daily activities, and continuation or escalation of gaming despite negative consequences.^6^ Consistent with the DSM-5 definition, the ICD-11 requires that these symptoms be evident for at least 12 months.

**Table 1:** Internet gaming disorder and gaming disorder criteria.

DSM-5 criteria for internet gaming disorder^a^	ICD-11 criteria for gaming disorder
Preoccupation with Internet games.	Impaired control over gaming.
Withdrawal symptoms when Internet gaming is taken away.	Increasing priority given to gaming to the extent that gaming takes precedence over other life interests and daily activities.
Tolerance—the need to spend increasing amounts of time engaged in Internet games.	Continuation or escalation of gaming despite the occurrence of negative consequences.
Unsuccessful attempts to control the participation in Internet games.	
Loss of interests in previous hobbies and entertainment as a result of, and with the exception of, Internet games.	
Continued excessive use of Internet games despite knowledge of psychosocial problems.	
Has deceived family members, therapists, or others regarding the amount of Internet gaming.	
Use of Internet games to escape or relieve a negative mood.	
Has jeopardized or lost a significant relationship, job, or educational or career opportunity because of participation in Internet games.	The behavior pattern is of sufficient severity to result in significant impairment in personal, family, social, educational, occupational or other important areas of functioning.

Abbreviations: DSM-5, Diagnostic and Statistical Manual of Mental Disorders, Fifth Edition; ICD-11, International Classification of Diseases, 11th Revision.

a A diagnosis of internet gaming disorder would require experiencing five or more of these symptoms within a year. The condition can include gaming on the internet with others or alone.

The diagnosis of disordered gaming has also been contentious due to high co-occurrence with other mental health concerns. Critics have argued that problematic gaming is better conceptualized as a maladaptive coping mechanism rather than a disorder unto itself, and that classifying problematic gaming as a mental disorder may spread moral panic and stigma.^13^ Moreover, the addiction model underlies the DSM-5 and ICD-11 criteria, yet alternate theoretical frameworks for gaming disorder have been proposed.^4^ However, these criticisms are not unique to disordered gaming: comorbidity is common across many psychiatric conditions,^14^ and maladaptive coping behaviors can develop into clinically significant disorders.^15^ Emerging evidence also suggests that for a subset of individuals, problematic gaming can be persistent and associated with functional impairment.^16^ Furthermore, framing disordered gaming as a source of “moral panic” risks understating the clinically significant impairment experienced by affected individuals, as diagnostic frameworks are intended to delineate thresholds of disorder rather than pathologize normative behavior.^17^ Taken together, these perspectives highlight ongoing debate while supporting the need for continued empirical investigation and nuanced conceptualization of disordered gaming.

## Methods

### Protocol and registration

The data reported are presented in accordance with the guidance of the Preferred Reporting Items for Overviews of Reviews (PRIOR).^18^ A formal protocol was retrospectively registered with the International Platform of Registered Systematic Review and Meta-analysis Protocols (INPLASY; INPLASY202670067; https://doi.org/10.37766/inplasy2026.7.0067). We followed an adapted version of PRIOR guidance to structure the review process, including predefined eligibility criteria and systematic search procedures. While not all elements of the guideline were applied rigidly, these adaptations were implemented to enhance methodological rigor and transparency, and we outline our criteria, search strategy, and study selection below.

### Search strategy and selection criteria

We included peer-reviewed systematic reviews and meta-analyses examining problematic gaming, gaming disorder, and/or internet gaming disorder. Studies were categorized into six thematic areas: assessment tools, epidemiology and prevalence, risk and maintenance factors, health outcomes, mental health correlates, and prevention and treatment approaches. Eligible studies primarily focused on adolescents and young adults. No language restrictions were applied. We excluded studies that did not meet these population or study design criteria or did not report relevant outcomes.

We conducted systematic searches in PubMed, Web of Science, and PsycInfo for articles published between September 1, 2016, and May 26, 2026. Inclusion criteria and detailed search strategies, including specific terms and Boolean operators, are provided in Supplemental Figures S1–S6. A PRISMA flowchart diagram with an overview of study inclusion across the six thematic searches is provided in Fig. 1.

**Fig. 1:**
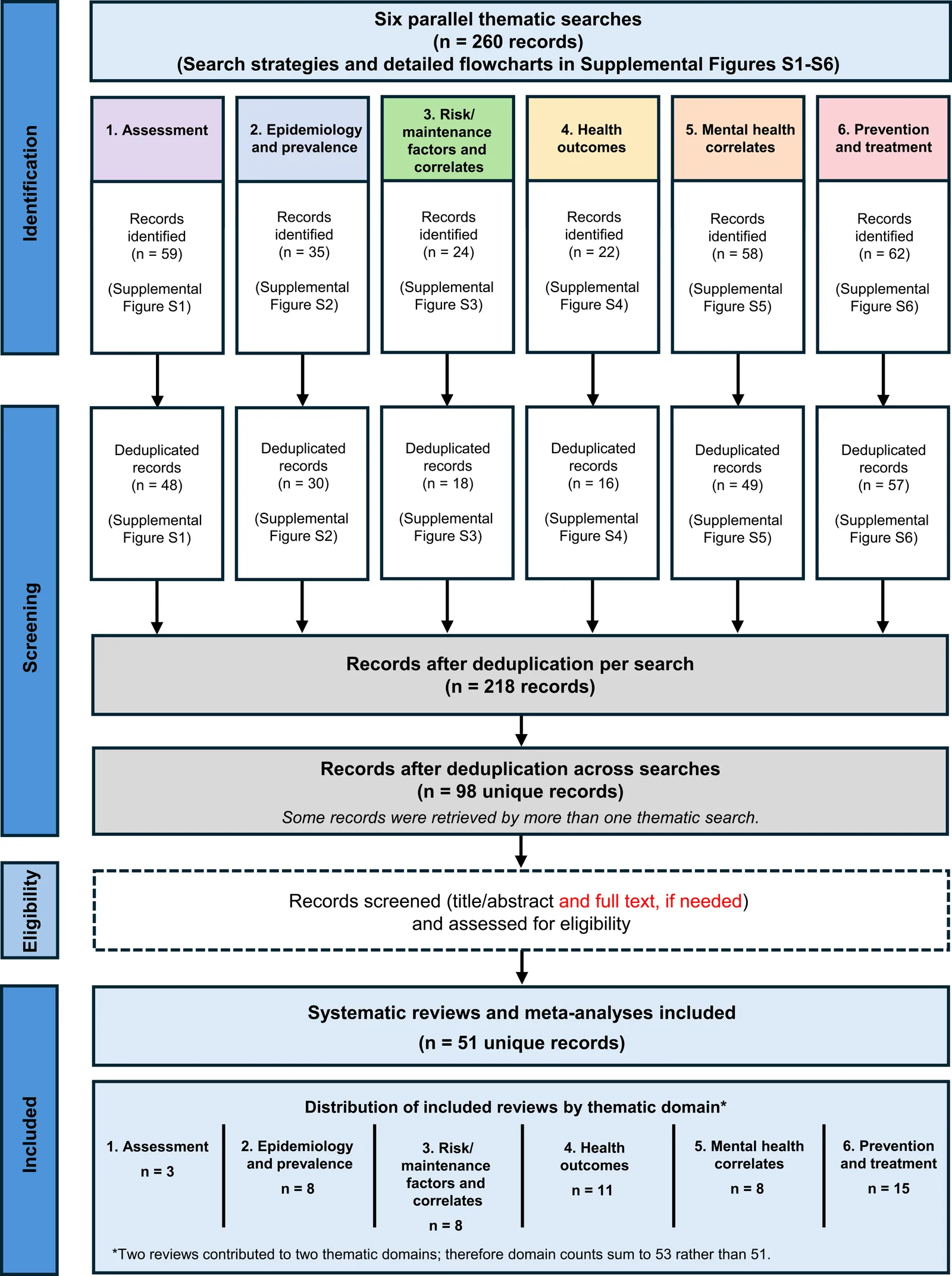
Overview of study identification and inclusion across six thematic searches.

All search results were independently screened by at least two reviewers per section. Initial searches were completed by Jason Nagata (JN), Stephanie Zhang (SZ), and Sahana Nayak (SN), with subsequent searches conducted by Christiane Helmer (CH). Following title and abstract screening, full texts of potentially eligible studies were retrieved and independently assessed for inclusion by section-specific reviewers (assessment tools: Kevin Handoko [KH], epidemiology and prevalence and risk/maintenance factors: SN; health outcomes: Kevin Bao [KB]; mental health correlates: CH; and treatment/prevention: SZ) and a second independent reviewer. Discrepancies were resolved through discussion, with JN serving as the adjudicator when consensus could not be reached.

### Risk of bias and quality assessment

We extracted and reported risk of bias and quality assessments as conducted within the included systematic reviews and meta-analyses. Initial extraction was completed by KH and SZ and verified by CH, KB, and SN. Where available, the assessments for each study were summarized to provide context for the strength and limitations of the synthesized evidence in Table 2. Quality appraisal summaries and narrative certainties for each thematic area (e.g., assessment, epidemiology and prevalence) are detailed in Supplemental Table S1.

**Table 2:** Characteristics, findings, and quality appraisal of included studies.

Authors, date	Type of study	Countries	Search range	Number of studies	Main results	Quality assessment and appraisal summary
Assessment						
Bernaldo-de-Quirós 2020^19^	Systematic review	United Kingdom (UK), Hungary, The Netherlands	2012–2018	13 studies	Out of the seven internet gaming disorder (IGD) assessments reviewed (IGD-20, IGDS9-SF, POGQ, POGQ-SF, VAT, C-VAT 2.0, IGD Scale), IGDS9-SF was the most widely used, with the most translations and validations.	Not included
King 2020^20^	Systematic review	Spain, UK, The Netherlands, South Korea, Germany, Hungary, Canada, United States (US), Sweden, Australia, Turkey, Switzerland	Up to 4/2019	320 studies	There are many conceptually similar tools, with no tool considered a gold standard. The tools with the most support include GAS-7, IGDT-10, IGDS9-SF, and IGD-9.	Not included
Chang 2023^21^	Systematic review	Indonesia, Germany, China, South Korea, Turkey, France, Italy, Iran, Portugal, Hungary, Malaysia, Finland, Norway, Ireland, Brazil, India, Australia, US, UAE, Hong Kong, Spain, Slovenia, UK, Saudi Arabia, Israel, Japan, multi-national	Up to 1/2022	93 studies	IGDS9-SF was the most commonly used assessment tool. Of the biopsychosocial factors in gaming disorder (GD) assessment tools, impaired self-concept, comorbidities, emotional dysregulation, and poor academic performance were consistently confirmed in GD, while reward activities, impulsivity, social relations, and family relationships showed mixed results.	The quality of articles was assessed through the Kmet quality checklist. The majority of articles scored over 0.9/1.0, indicating high quality.
Epidemiology and Sociodemographics						
Stevens 2021^22^	Systematic review and meta-analysis	Canada, China, Germany, Finland, Hong Kong, Hungary, Iran, Japan, Lithuania, The Netherlands, Norway, Sweden, Slovenia, South Korea, Switzerland, Taiwan, UK, US, multi-national	2009–2019	53 studies	The worldwide prevalence of gaming disorder was 3.05% (confidence interval: [2.38, 3.91]); this figure was adjusted to 1.96% [0.19, 17.12] when considering only studies that met more stringent sampling criteria. Rates were 2.5:1 in favor of males. Regression analysis identified that adolescent samples tended to report higher prevalence estimates.	Potential publication bias was assessed using Rosenthal's fail-safe N. No indication of bias was observed (fail-safe N = 12,350), suggesting that 12,350 null studies would be required to meaningfully alter the estimated prevalence.
Gou 2024^23^	Systematic review and meta-analysis	China	Up to 4/2024	43 studies	Prevalence of IGD among Chinese adolescents was 10% (95% confidence interval: 8%–10%) across 43 cross-sectional studies. Rates were higher in males at 17% (CI: 13%–21%). Gender, sample size, and survey year were significant moderators.	The cross-sectional study quality assessment by the Agency for Healthcare Research and Quality (AHRQ) was used to evaluate studies. Studies were categorized as low-quality with scores ranging from 0 to 3, medium-quality with scores from 4 to 7, and high-quality with scores from 8 to 11. Low-quality studies were excluded from this analysis.
Mihara 2017^24^	Systematic review	UK, Norway, Germany, The Netherlands, France, Hungary, Spain, Finland, Denmark, Sweden, US, Singapore, South Korea, China, Australia, Greece, Iceland, Romania, Poland, Canada, Estonia, Israel, Italy, Slovenia	Up to 5/2016	50 studies	Prevalence of IGD across 37 cross-sectional studies ranged from 0.7% to 27.5%, with male prevalence (2.0%–22.7%) consistently exceeding female prevalence (0%–4.9%) across nearly all studies. Adolescent samples ranged from 1.3% to 19.9%, comparable to mixed-age samples (0.3%–27.5%). Longitudinal findings suggested IGD symptoms were often unstable over time.	Not included
Putra 2023^25^	Systematic review	China, Iran, Taiwan, Indonesia, Australia	2019–2021	5 studies	7391 participants aged 12.34–20.60 years were included. Internet addiction prevalence could not be pooled due to inconsistent instrumentation. Of two studies assessing online gaming disorder via IGDS9-SF, one found significantly higher prevalence during COVID-19 than before (p = 0.025). All studies reported associations with depression, anxiety, stress, and sleep disorders.	Newcastle Ottawa Scale (NOS), NOS score of 7
Gisbert-Pérez 2026^26^	Systematic review and meta-analysis	India, Saudi Arabia, Jordan, Syria, Kuwait, Spain, US, Italy, Mexico, Germany, Sweden, Australia, China, South Korea, Egypt, Tunisia, Lebanon, Switzerland, Malaysia, Philippines, Israel, France, Norway, Canada, Bangladesh, Japan, Thailand, Nepal, Indonesia, Greece, Iran, United Arab Emirates, Macao, Hong Kong, Taiwan	Up to 5/2025	93 studies	Across 96 analytical samples (n = 149,601), IGD prevalence in young adults was 6.1% (95% confidence interval: 5.03%–7.40%), ranging from 0% to 63% across primary studies. Gamer-only samples yielded higher prevalence than mixed samples. Larger sample sizes and higher proportions of female participants were associated with lower prevalence.	Study quality was assessed using the Joanna Briggs Institute (JBI) checklist: 39.6% were low quality (n = 38), 51% moderate (n = 49), and 9.4% high (n = 9). Publication bias was evaluated using the DOI plot and LFK index, which showed marked asymmetry (LFK = −3.8), indicating likely bias. However, heterogeneity remained stable, suggesting the findings are robust and not driven by outliers.
Satapathy 2024^27^	Systematic review and meta-analysis	Australia, Brazil, China, Croatia, Egypt, Finland, Germany, India, Indonesia, Iran, Ireland, Israel, Kyrgyzstan, Korea, Lebanon, Malaysia, Netherlands, Norway, Saudi Arabia, Slovenia, Spain, Sweden, Taiwan, Tunisia, Turkey, US, Vietnam	2010–2023	84 studies	The pooled prevalence of gaming disorder was 8.6% (95% CI, 6.9–10.8%).	Quality assessment was performed using the JBI tool. Two reviewers independently performed data extraction to ensure reliability. 43 studies were assessed for quality: 5 were classified as low, 27 as moderate, and 11 as high.
Limone 2023^28^	Systematic review and meta-analysis	US, Sweden, France, Italy, Ireland, China, Lebanon, Brazil, Norway, Turkey, Czech Republic, Finland, India, Ghana, Saudi Arabia, Spain, Taiwan, International	2017–2022	12 studies	The pooled prevalence level of gaming addiction was 5.0% (95% CI, 2.1–8.8%).	Quality of evidence was assessed through the adopted critical appraisal tool provided in the Best Bets Critical Appraisal Worksheets. For the majority of studies, approximately half of the assessment items were clearly stated.
Chia 2020^29^	Meta-analysis	Southeast Asian countries, including Brunei, Myanmar, Cambodia, Timor-Leste, Indonesia, Laos, Malaysia, Philippines, Singapore, Thailand, and Vietnam	Up to 1/2020	24 studies	The pooled prevalence rate for Internet addiction and gaming disorders was 20.0% (95% CI 14.5%–27.0%) and 10.1% (95% CI: 7.3%–13.8%), respectively.	There was no evidence of significant publication bias, as shown by a non-significant Egger's regression test.
Risk/Maintenance Factors and Correlates						
Gao 2022^30^	Systematic review and meta-analysis	Germany, China, Japan, Korea, France, US, Spain, Brazil, Turkey, Hungary, Saudi Arabia, Thailand, Iran, Finland, Egypt, India, Canada, Switzerland, Greece, The Netherlands, Mexico, Norway, Pakistan, Lebanon, Italy, Australia, Slovenia, Sweden, Singapore, Austria, England	Up to 5/2021	37 studies	Stress, long average game time, family dysfunction, poor academic performance, being bullied, bullying, interpersonal problems, hyperactivity/inattention, anxiety, depression, emotional distress and low self-esteem are possible risk factors for IGD among Adolescents and Young Adults (AYAs).	Study quality was evaluated using Agency for Healthcare Research and Quality (AHRQ) and standard checklist criteria. Funnel plot asymmetry and both Begg's and Egger's tests (p < 0.001) indicated potential publication bias, which was adjusted using the trim-and-fill method. The pooled IGD prevalence increased from 9.9% (95% CI: 8.6%–11.3%) to 13.1% (95% CI: 11.6%–14.6%) after adjustment.
Schneider 2017^31^	Systematic review	Singapore, Korea, Taiwan, China, Hong Kong, Germany, Australia, Sweden, Turkey	2007–2016	14 studies	Poorer quality of the parent–child relationship was associated with increased severity of problem gaming. Both maternal and paternal relationships were negatively associated with problem gaming, with longitudinal evidence indicating that the paternal bond in particular was a protective factor against problem gaming.	Not included
Lukavská 2022^32^	Meta-analysis	South Korea, Malaysia, Bangladesh, Taiwan, China, Singapore, Indonesia, Spain, Slovakia, Germany, Greece, The Netherlands, Czech Republic, Romania, Iran, Turkey, Israel, US	Up to 4/2022	41 studies	Parental warmth showed a small negative association with problematic internet use, with stronger effects in younger and mixed-age samples and for GD specifically. Parental control and authoritative parenting showed small negative associations. Active mediation showed inconsistent associations, with no significant moderators. Restrictive mediation showed no meaningful association overall.	Risk of bias assessment was based on ROBUST, which specified 8 criteria relevant for survey studies. Each study could reach 0 to 8 points where 0 is the lowest quality (not meeting any criteria) and thus high risk of bias; and 8 is the highest quality (meeting all criteria) and low risk of bias. Scores of included studies ranged from 3 to 7.
Sugaya 2019^33^	Systematic review	Germany, South Korea, Australia, Slovenia, Norway, Estonia, Italy, Romania, Spain, Greece, Iceland, The Netherlands, Poland, Sweden, Hungary	Up to 2/2018	51 studies	Poor parent–child relationships—particularly weak paternal bonds—family influence, lack of parental supervision, early gaming experiences, peer pressure, access to internet cafes, and aggressive promotional activities at gaming venues were associated with problematic gaming. Family influence and supervision, peer pressure, accessibility of gaming, and early exposure to gaming environments were associated with IGD. Family and peer influences, early gaming exposure, and the centrality of gaming in daily life, including its use as a compensatory activity or a focus of one's identity, were associated with GD.	Not included
Petrescu 2025^34^	Systematic review	China, Korea, Iran, Taiwan, India, Thailand, Vietnam, Singapore, Turkey, Germany, Italy, Spain, Denmark, The Netherlands, Romania, Finland, France, Belgium, Brazil, Australia	2011–2025	54 studies (48 cross-sectional, 16 longitudinal)	Protective factors included parental warmth, communication, and parent-child closeness. Risk factors included controlling, permissive, and hostile parenting, familial disharmony, and lower socioeconomic status. Parental mediation showed inconsistent longitudinal effects.	Methodological quality has been assessed using an adapted version of the Downs and Black (1998) checklist. Only 10 of the original 27 items were selected, as they were representative of this review's studies. Longitudinal studies had score of at least 6.
Nielsen 2020^35^	Systematic review	South Korea, China, Germany, Singapore, Hong Kong, Spain, Taiwan, Australia, France, Iran, Italy, Turkey	2004–2019	26 studies	31,853 adolescents were surveyed for problematic gaming, and all were sampled via schools. The prevalence of problematic gaming ranged from 0.8% to 30.4%.	Methodological quality was assessed using a modified 10-item checklist based on sample size, longitudinal design, sampling method, and low attrition. Evaluation considered the completeness of demographics and psychometric validity of instruments. The problematic gaming study quality score was low, with a range of 9–15 (median 12.0) out of a maximum possible score of 20.
Atalan Ergin 2025^36^	Systematic review	Brazil, Czech Republic, European Countries, Finland, France, Germany, Israel, Italy, Netherlands, Romania, Slovenia, Spain, Turkey, Iran, China, Hong Kong, Singapore, Australia, Korea, Vietnam	1995–2024	77 studies	Positive family functioning was associated with low IGD levels while negative family functioning predicted higher risk. Additional risk factors include neglectful or abusive parenting, nontraditional family type (separated or single-parent), and parental mental health.	Not included
Coşa 2023^37^	Meta-analysis	Iran, Belgium, Germany, Greece, Hungary, Norway, France, Slovakia, Czech Republic, South Korea, China, Taiwan, Singapore, China, Turkey, Spain, Italy, The Netherlands, Hong Kong, Sweden	Up to 1/2021	38 studies	Identified protective parental behaviors include giving autonomy to young people, providing a positive environment, and age-appropriate monitoring. Harmful behaviors include aversiveness, overinvolvement, and withdrawal. Withdrawal and overinvolvement showed the strongest link with IGD.	Methodological quality was assessed using the NIH Quality Assessment Tool for Observational Cohort and Cross-Sectional Studies, with a maximum score of 14. 7 studies were rated high quality with more than 10 points, 22 studies as fair with 7–10 points, and 9 were inadequate with less than 7 points. Publication bias was assessed using a funnel plot and no small study effects were identified.
Health Outcomes						
Khor 2023^38^	Systematic review	South Korea, China	2013–2021	24 studies	GD is associated with neurobiological correlates assessed by EEG, fMRI, sMRI, and proton MRS that are comparable with substance addiction and associated with differences in emotion regulation, inhibition, self-identity, and several domains of cognitive functioning.	Methodological quality was assessed using JBI's Critical Appraisal Tools. Specific details about quality of studies included were not explained in the review.
Kristensen 2021^39^	Systematic review and meta-analysis	France, Turkey, Saudi Arabia, Egypt, Norway, Italy, Iran, India, Indonesia, Malaysia, Mexico, Philippines, Spain, Lebanon, South Korea, Australia, Taiwan, China, Finland, Japan, Portugal, Germany, Singapore, Brazil, US, Hong Kong, UK	2009–2021	33 studies	Problematic gaming is associated with shorter sleep duration, poorer sleep quality, higher daytime sleepiness, and increased sleep problems.	Methodological quality was assessed using Newcastle–Ottawa Quality Assessment Scale adapted for cross-sectional studies. This scale assesses the sample selection process, comparability between groups, and ascertainment of results and has a range of 0–9, with higher scores being greater quality. The scores of included studies ranged from 2 to 9, with a mean of 5.5 and standard deviation of 1.5. Interrater reliability for this scale was good.
Benchebra 2019^40^	Critical review/Systematic review	US, Sweden, UK, Canada, Japan, Singapore, China, Taiwan, Germany, France, Norway, Finland	Up to 9/2017	25 studies: 14 studies for gambling, 11 studies for video gaming	GD is associated with sleeping problems, joint and muscle pain, headache, and vision problems. Symptoms were most frequently reported by teenagers, and the most frequently reported symptom was sleeping problems.	Not included
Sugaya 2019^33^	Systematic review	Germany, South Korea, Australia, Slovenia, Norway, Estonia, Italy, Romania, Spain, Greece, Iceland, The Netherlands, Poland, Sweden, Hungary	Up to 2/2018	51 studies	Adolescents with IGD had differences in the prefrontal cortex and striatum that are associated with impaired cognitive control functions and impulsivity.	Not included
Zhu 2025^41^	Systematic review	South Korea, China	2015–2025	21 studies	IGD is associated with reduced functional connectivity in fronto-striatal circuits and dysregulation of default mode network, executive control network, and salience network, which are involved in attention and cognitive control. IGD is also associated with reduced hemispheric synchronization, reduced fractional anisotropy in corpus callosum, and differences in regional activity and coordination. IGD is fundamentally a disorder of reward system dysfunction with a clear neurobiological basis.	Methodological quality was rated by scoring low, uncertain, or high risk of bias across several domains: selection of participants, inter-group comparability, control for confounding/comorbidities, standardization of fMRI acquisition, blinding of outcome assessment, and overall risk of bias. In summary, although most studies demonstrated good control over key methodological aspects, limitations in inter-group comparability, confounding control, and implementation of blinding may somewhat affect the robustness of the conclusions drawn in this study.
Schettler 2022^42^	Systematic review	South Korea, China	2010–2020	21 studies	Alterations in connectivity and neural efficiency in prefrontal, temporoparietal, frontolimbic, subortical regions is associated with problematic gaming, which supports model of cognitive control and affective imbalance model.	Not included
Yan 2021^43^	Meta-analysis	South Korea, China, Australia	Up to 11/2020	25 studies	IGD is associated with differences in functional connectivity within and between different resting state networks, the default mode network, ventral attention network, sensorimotor network, limbic network, and frontoparietal network. These differences may underlie impaired self-monitoring, attentional bias towards incentive triggers, development of habitual behaviors, heightened motivation towards gaming cues, and difficulties inhibiting addictive behaviors.	Jackknife sensitivity analysis was conducted, only ventral attention network-sensorimotor network extent-based thresholded effect not replicable.
Che Mokhtar 2025^44^	Systematic review	Turkey, The Netherlands, China, South Korea, Iran	2010–2020	14 studies	Internet addiction was significantly associated with higher BMI and in adolescents. Gaming addiction was also associated with obesity, emotional eating, and poorer eating habits.	Methodological quality was assessed using JBI Critical Appraisal Tools, which apply design-specific criteria. The first author conducted all appraisals, with verification by a co-author. Studies were rated as high (>70% “Yes”), medium (50–70%), or low (<50%). All studies were classified as high quality; three had two “No” ratings, and three had one.
Zheng 2019^45^	Meta-analysis	China, Taiwan, South Korea, Germany, The Netherlands	2008–2018	40 studies	IGD is associated altered with hyperactivation and hypoactivation in several regions within in key brain networks involved in attention, executive control, and risk decision making.	The jackknife sensitivity analyses and Egger's test were conducted and did not reveal publication bias in this meta-analysis.
Cherif 2025^46^	Systematic review	South Korea, Turkey	2018–2023	4 studies	IGD is associated with impaired phasic parasympathetic reactivity during game engagement.	Methodological quality of studies was independently assessed by the authors using Risk of Bias in Non-randomized Studies (ROBINS-I) tool, which evaluates confounding, selection of participants, classification of exposures, deviations from intended exposures, missing data, measurement of outcomes, and selection of the reported result. All 4 studies were classified as low risk of bias.
Lemos 2014^47^	Systematic review	China, South Korea, Germany, Taiwan, The Netherlands, Europe	Up to 7/2013	16 studies	Video game addiction was associated with structural and functional changes in the frontal, parietal, and temporal lobes, as well as the basal ganglia, thalamus, insula, and cerebellum. Neural changes are similar to those found in substance and internet addiction.	Not included
Mental Health Correlates						
Pallavicini 2022^48^	Systematic review	UK, US, Sweden, Vietnam, Italy, Turkey, Spain, Malaysia, South Korea, Germany, Austria, Switzerland, Japan, Hong Kong, China	2019–2022	24 studies	During the COVID-19 pandemic, GD was associated with maladaptive coping regulatory styles, poor social support, poor mental health, academic stress, and excessive use of social networks. Depressive and anxiety symptoms before the COVID-19 pandemic positively predicted IGD during the COVID-19 pandemic.	Mixed Methods Appraisal Tool (MMAT): 9 scored 100 (highest quality), 13 scored 75, and 2 scored 50
Coutelle 2024^49^	Systematic review	South Korea, China, Saudi Arabia, Turkey, Philippines, Taiwan, Germany, Hungary, Ireland, Italy, The Netherlands, Norway, Portugal, Spain, France, Egypt, US	2013–2024	30 studies	There is a high prevalence of attention-deficit/hyperactivity disorder (ADHD) among adolescents with problematic gaming, along with associations between gaming addiction and inattention, anxiety, depression, and behavioral and emotional problems.	National Institute of Health's Quality Assessment Tool for Observational Cohort and Cross-Sectional Studies: The mean quality score was 8.21 (SD = 2.06)
Putra 2023^25^	Systematic review	China, Iran, Taiwan, Indonesia, Australia	2019–2021	5 studies	During the COVID-19 pandemic, GD was significantly associated with higher levels of depression, anxiety, stress, and insomnia, as well as lower quality of life and subjective well-being.	NOS score of 7
Ghali 2023^50^	Systematic review	South Korea, Japan, The Netherlands, Turkey, US, Russia, Austria, France, Canada, Spain, Taiwan, Norway	2018–2023	20 studies	IGD is bidirectionally associated with depression and is linked to declines in psychological well-being, mood dysregulation, life satisfaction, and academic performance.	NOS for observational studies and the Scale for the Assessment of Narrative Review Articles (SANRA) for traditional reviews. 20 articles were identified as meeting the criteria of medium or high-standard quality and subsequently included in the systematic review.
Richard 2020^51^	Systematic review	US, Canada, China, Australia, Italy, Sweden, South Korea, Singapore, Spain, Germany, Norway, France, Belgium, Turkey, Finland, The Netherlands	1994–2019	71 studies	Both problem gambling and gaming are consistently concurrently associated with conduct problems and depressive symptoms.	Not included
Losaberidze 2025^52^	Systematic review	China, Turkey, UK, US, Taiwan, Israel, South Korea, Hungary, Croatia, Norway, Europe	Up to 4/2025	40 studies	In students and adolescents, the severity of IGD or problematic gaming was linked to higher risk of NSSI and suicidality, with evidence of anxiety also acting as a mediator and moderator in this relationship.	The JBI critical appraisal tools were used to assess the authenticity, relevance, and results of both quantitative and qualitative studies. Included studies passed at least 6 assessment checks.
Hu 2025^53^	Systematic review and meta-analysis	China, Singapore, India, South Korea, US, Slovenia, UK, Australia, Turkey, Iran	Up to 11/2023	40 studies	Lower self-control was associated with problematic gaming	Quality Assessment Tool for Systematic Reviews of Observational Studies, which consists of 10 items focusing on four aspects: introduction (objective), method (study design, representativeness, response rate, attrition rate, bias in measurement, and confounders), analysis (data analysis plan), and results (effect size and significance). Twenty-three studies were evaluated as “Good,” 22 as “Satisfactory,” and only 1 was rated as “Inadequate.”
Li 2023^54^	Meta-analysis	India, China, Turkey, Singapore, Pakistan, Switzerland, South Korea, The Netherlands, Palestine, Japan, Finland	1999–2022	24 studies	Higher levels of IGD may be associated with more aggression, and conversely, greater aggression may be linked to more higher levels of IGD.	Egger's regression test did not reveal a significant bias.
Prevention and Treatment						
Chang 2022^55^	Meta-analysis	Taiwan, US, Iran, China, South Korea	2000–2017	124 studies	Combination therapy with pharmacotherapy and either cognitive behavioral therapy (CBT) or multi-level counseling (MLC) was the most effective treatment modality of the five modalities studied (the other four: pharmacotherapy; cognitive behavioral therapy; multi-level counseling program; other methods including electro-acupuncture; other methods combined with CBT or MLC).	Risk of bias was evaluated via funnel plots and Egger's regression intercept tests for bias or Begg's test. Publication bias was found to be significant (t = 8.71, p-value <0.001 for Egger's test and z = 5.25, p < 0.001 for Begg's test).
Colder Carras 2024^56^	Rapid systematic review	Thailand, US, France, Iran, Italy, Germany, China, Spain, Turkey, UK, Palestine, Canada, Taiwan, Malaysia, Finland, Russia, Norway, Austria, South Korea	Up to 3/2023	41 studies	Most interventions were for primary prevention and took combined approaches, most often combining digital wellbeing education and self-regulation approaches. The reduction in average minutes of daily use was smallest for gaming time and screen time, averaging 18.9 min per day compared to 40.9 min on social media, 34.0 min per day of smartphone use, and 25.1 min per day of internet use.	All reviews related to gaming received an AMSTAR 2 score of “critically low”. All but one included controlled study reported statistical test results.
King 2014^57^	Systematic review	US, UK, China, Turkey, Taiwan, The Netherlands, Czech Republic, Greece, Spain, Singapore, South Africa, Switzerland	2000–2013	36 studies	Effectiveness not determined. Because the treatment studies did not report change in the criterion of preoccupation between baseline and post-intervention, it was not possible to make therapeutic conclusions about the 7 cognitive therapy or cognitive behavioral therapy interventions.	Not included
Park 2025^58^	Systematic review	Sweden, Germany, China, Philippines, Iran, Spain, South Korea, Norway, Thailand, Japan, Australia, Switzerland	2005–2025	30 studies	Interventions addressing GD, particularly those employing cognitive behavioral therapy alone or in combination with pharmacotherapy, show potential. However, effectiveness ascertainment is limited by studies' relatively short-term follow-up, lack of a control group, and lack of randomization.	Using the Effective Public Health Practice Project (EPHPP) Quality Assessment Tool for Quantitative Studies, most studies were rated as moderate quality, with fewer classified as strong or weak. Among Randomized Controlled Trials (RCTs), trial registration was uncommon and no protocols were published; only one non-RCT was registered.
Harpas 2025^59^	Systematic review and meta-analysis	China, South Korea, Thailand, US, Turkey, Sweden, Philippines, Switzerland	2007–2024	21 studies	Interventions showed a large reduction in GD symptoms in adolescents, which remained significant at three months. Gaming time also decreased both immediately after treatment and at follow-up across adolescents and adults. Most interventions used CBT, though its effectiveness did not differ significantly from other approaches.	Using the Risk of Bias 2 (RoB 2) tool, most studies were low risk for protocol deviations, missing data, and outcome measurement. Reporting bias was generally low or raised some concerns. Overall, three studies were low risk, four were high risk, and most had some concerns.
King 2018^60^	Systematic review	South Korea, China or Hong Kong, Germany, US, Spain, South Africa	2007–2016	13 studies	Findings were mixed and warrant further empirical study. South Korea's governmental model stood out for its coordination and long-term multilevel strategic plans. In contrast to the USA and Australia, East Asian models of prevention formally recognize problematic gaming use as a disorder.	Not included
Núñez-Rodríguez 2025^61^	Systematic review	South Korea, China, Turkey, Philippines, Sweden, Switzerland, Spain, Thailand, Japan, US	2014–2025	22 studies	CBT in combination with physical exercise or mindfulness techniques showed the greatest efficacy in reducing symptoms of IGD. CBT was the most effective isolated intervention. All interventions studied, including virtual reality and transcranial direct current stimulation, showed effectiveness.	Methodological quality assessment using the critical appraisal tools of the Joanna Briggs Institute at the University of Adelaide, adapted to each study's design. Most studies achieved high scores, consistently surpassing the established threshold for methodological quality and risk of bias.
Lampropoulou 2022^62^	Systematic review	China, South Korea, Norway, Thailand, Japan, Germany, Spain, Taiwan	2010–2022	16 studies	CBT has demonstrated effectiveness, but more research is needed in adolescent populations with comorbidities. Combination therapy with CBT and pharmacotherapy may be recommended for adolescents with IGD and comorbid conditions.	Using the NOS, 11 studies were rated as high-quality and the other five as mediocre quality.
Ock 2025^63^	Systematic review and meta-analysis	Germany, Nigeria, China, South Korea, US, Turkey, Switzerland	Up to 5/2024	18 studies	Non-pharmaceutical interventions, specifically CBT, multidimensional family therapy, and virtual reality-based trainings, are effective in treating GD. However, the efficacy is more pronounced in young adults compared to adolescents (g = −1.14 vs −0.45).	Using the Cochrane RoB2 tool, four RCTs were rated low risk while three were high risk overall. Evidence of publication bias was observed, and adjustment methods suggested the true effect may be stronger than initially estimated.
Ayub 2023^64^	Systematic review	China, Germany, South Korea, Turkey, Switzerland	2008–2023	10 studies	CBT was effective in reducing symptoms of IGD. Pharmacotherapy was effective in adolescents with ADHD as a comorbidity. School-based education was effective in reducing computer gaming use.	Using the Cochrane risk of bias tool, two studies were deemed as high risk, one as moderate to high risk, two as low risk, three as some concerns, and 2 as unclear.
Király 2018^65^	Systematic review	Thailand, Vietnam, South Korea, China, Western countries (e.g., Germany), International	2000–2016	12 studies	Policies were grouped into three categories: restricting game access (e.g., shutdown policies, fatigue systems, parental controls), reducing harm (e.g., warning messages), and providing support services. However, many of these measures have been either less effective than expected or insufficiently evaluated for efficacy.	Not included
Aryani 2024^66^	Systematic review	South Korea, Turkey	Up to 2/2024	3 studies	Physical exercise was found to be an effective intervention to reduce gaming addiction severity. Possible mechanisms include regulatory effects on the hypothalamic-pituitary-adrenal axis, release of brain derived neurotrophic factor, and reducing gaming duration.	Using the Revised Cochrane risk-of-bias tool, one study was considered good quality while the other 2 have some concerns.
Gioia 2019^67^	Systematic review	Hong Kong, China, US, South Korea, Australia, Italy, Germany, Spain, Japan, Malaysia	Up to 7/2018	20 studies	Cognitive behavioral therapy, family programs, and pharmacological therapies have been utilized as treatments for internet addiction and IGD. Conflicting findings and heterogeneity of definitions of internet addiction and IGD limit conclusions on effective treatment.	Not included
te Wildt 2025^68^	Systematic review	South Korea, US, Germany, China, Spain, Denmark	1996–2022	30 studies	Cognitive-behavioral psychotherapy is the recommended first choice treatment, while addressing comorbidities such as depressive disorders, anxiety disorders, and ADHD. Bupropion is the only pharmacologic treatment justifiable in the absence of comorbidities.	Not included
Lo 2023^69^	Meta-analysis	Palestine, Israel, Iran, Germany, South Korea, Turkey, China, Nigeria, Philippines, US, Thailand, Switzerland, Spain, Austria	Up to 3/2022	34 studies	Psychosocial interventions for Internet addiction severity were found to have a significant effect with a large pooled effect size (Cohen's d = 1.790; 95% CI [1.365, 2.214], p < 0.001). The effect was largest immediately following intervention, with a decrease at 1-month follow-up, and partial rebound at 3 and 6 months.	Quality of included studies was assessed using the 25-item Consolidated Standards of Reporting Trials (CONSORT) checklist, up to a score of 25. The scores ranged from 10 to 23, with an average of 14.35. Funnel plots and the Egger's test were used to estimate publication bias, and the significant result of the Egger's test (p < 0.001) implicates presence of publication bias.

Abbreviations: ADHD, attention-deficit/hyperactivity disorder; AHRQ, Agency for Healthcare Research and Quality; AMSTAR 2, A MeaSurement Tool to Assess systematic Reviews, version 2; AYA, adolescent and young adult; BMI, body mass index; CBT, cognitive behavioral therapy; CI, confidence interval; CONSORT, Consolidated Standards of Reporting Trials; C-VAT 2.0, Clinical Video game Addiction Test 2.0; EEG, electroencephalography; EPHPP, Effective Public Health Practice Project Quality Assessment Tool for Quantitative Studies; fMRI, functional magnetic resonance imaging; GAS-7, 7-item Gaming Addiction Scale; GD, gaming disorder; IGD, internet gaming disorder; IGD-9, 9-item dichotomous Internet Gaming Disorder Scale; IGD-20, Internet Gaming Disorder Test–20; IGDS9-SF, Internet Gaming Disorder Scale–Short-Form; IGDT-10, Ten-Item Internet Gaming Disorder Test; JBI, Joanna Briggs Institute; LFK, Luis Furuya-Kanamori; MLC, multi-level counseling; MMAT, Mixed Methods Appraisal Tool; MRS, magnetic resonance spectroscopy; NIH, National Institutes of Health; NOS, Newcastle–Ottawa Scale; NSSI, nonsuicidal self-injury; POGQ, Problematic Online Gaming Questionnaire; POGQ-SF, Problematic Online Gaming Questionnaire–Short Form; RCT, randomized controlled trial; RoB, risk of bias; RoB 2, Cochrane Risk of Bias 2 tool; ROBINS-I, Risk of Bias in Non-randomized Studies; ROBUST, Risk Of Bias in non-randomized Studies of exposures for Use in Systematic reviews of Transversal studies; SANRA, Scale for the Assessment of Narrative Review Articles; SD, standard deviation; sMRI, structural magnetic resonance imaging; UAE, United Arab Emirates; UK, United Kingdom; US, United States; VAT, Video game Addiction Test.

To quantify overlap among the included systematic reviews and meta-analyses, we calculated the corrected covered area (CCA) index. Because the six thematic domains addressed distinct research questions and bodies of literature, CCA was calculated separately for each domain.

### Role of the funding source

The funders had no involvement in study design, data collection, data analyses, data interpretation, or the writing of the report.

## Results

Our search yielded a total of 98 unique records, of which 51 unique reviews met inclusion criteria following title/abstract screening and full-text assessment. Of the included reviews, 3 focused on assessment tools, 8 on epidemiology and prevalence, 8 on risk and maintenance factors, 11 on health outcomes, 8 on mental health correlates, and 15 on prevention and treatment approaches, with 2 reviews overlapping across two sections. Study characteristics, key findings, and quality assessments are summarized in Table 2. Of the 51 included reviews, 30 (59%) conducted a quality assessment. Narrative certainty across thematic domains ranged from low to low-moderate (Supplemental Table S1). The Joanna Briggs Institute (JBI) tools were the most frequently used (6 reviews) for quality assessment, followed by the Newcastle Ottawa Scale (NOS) and Cochrane Risk of Bias (RoB) variants (4 each). None of the reviews used the Grading of Recommendations Assessment, Development, and Evaluation (GRADE) framework for quality assessment.

Overlap among included reviews was generally low. CCA indices indicated slight overlap for epidemiology and sociodemographics (CCA = 0.032), health outcomes (CCA = 0.047), mental health correlates (CCA = 0.018), and treatment and prevention (CCA = 0.040), moderate overlap for risk and maintenance factors (CCA = 0.088), and high overlap for assessment studies (CCA = 0.138), reflecting the shared psychometric validation literature across assessment reviews.

## Assessment

Numerous measures have been developed to assess the criteria for disordered gaming and problematic video game use more broadly. The Video Game Addiction Questionnaire, Gaming Addiction Scale-7, 9-item dichotomous Internet Gaming Disorder Scale, Young Diagnostic Questionnaire, and Compulsive Internet Use Scale-14 cover a subset of the criteria in DSM-5 and ICD-11.^20^ Supplemental Table S2 summarizes the coverage of the DSM-5 and ICD-11 criteria of selected tools. Some are tools to screen for clinical internet gaming disorder (e.g., 9-item dichotomous Internet Gaming Disorder Scale), while others are continuous measures of the severity of problematic gaming (e.g., Video Game Addiction Questionnaire). Table 3 provides a description of selected tools, including their components, scoring, and clinical cutoffs.

**Table 3:** Selected questionnaires for evaluating problematic video gaming.

Questionnaire	Description	Rating scale	Validated populations	Full alignment with definitions	Clinical cutoff	Year first described	Country of development
Young Diagnostic Questionnaire (YDQ)	8-item questionnaire adapted from the DSM-IV's definition of pathological gambling	“Yes” or “No"	Adolescents and Adults	None	5/8	1998	United States
Compulsive Internet Use Scale (CIUS-14)	14-item unidimensional questionnaire including five domains (loss of control, preoccupation, withdrawal symptoms, coping, and conflict)	5-point Likert-type scale (0 = “Never” to 4 = “Very Often")	Adolescents and Adults	None	28/56	2009	Netherlands
7-item Gaming Addiction Scale (GAS-7)	7-item questionnaire measuring salience, tolerance, mood modification, withdrawal, relapse, conflict, and problems	5-point Likert-type scale (1 = “Never” to 5 = “Very Often")	Adolescents and Adults	None	At least 4 responses 3 or higher	2009	Netherlands
Internet Gaming Disorder Scale-Short Form (IGDS9-SF)	9-item questionnaire based on 9 criteria of DSM-5 definition of IGD	5-point Likert-type scale (1 = “Never” to 5 = “Very Often")	Adolescents and Adults	DSM-5 & ICD-11	36/45	2015	United Kingdom
9-item dichotomous Internet Gaming Disorder Scale (IGD-9)	9-item questionnaire based on 9 criteria of DSM-5 definition of IGD	“Yes” or “No"	Adolescents and Adults	None	5/9	2015	Netherlands
Personal Internet Gaming Disorder Evaluation-9 (PIE-9)	9-item questionnaire based on the 9 criteria of DSM-5's IGD	5-point Likert-type scale (1 = “Never” to 5 = “Very Often")	Adolescents and Adults	DSM-5 & ICD-11	5/9	2016	Australia
Ten-Item Internet Gaming Disorder Test (IGDT-10)	10-item questionnaire based on the 9 criteria of DSM-5's IGD	3-point Likert-type scale (0 = “Never” to 2 = “Often")	Adolescents and Adults	DSM-5 & ICD-11	5/9 must be “Often"^a^	2017	Hungary
Gaming Disorder Scale—Family (GDS-F)	18-item questionnaire for family members, based on the 9 criteria of DSM-5's IGD	5-point Likert-type scale (1 = “Never” to 5 = “Very Often")	Adolescents	DSM-5	5/9 as “Very Often”	2021	Turkey
Development of the DSM–5 IGD Symptoms Checklist for Adolescents (DISCA)	9-item questionnaire based on the 9 criteria of DSM-5's IGD	“Yes” or “No"	Adolescents	DSM-5	N/A	2021	China
Videogame Addiction Questionnaire (VGAQ)	6-item questionnaire adapted from Bergen Facebook Addiction Scale	6-point Likert-type scale (1 = “Never” to 6 = “Very Often")	Adolescents	None	N/A	2022	United States

Abbreviations: CIUS-14, Compulsive Internet Use Scale-14; DISCA, DSM-5 IGD Symptoms Checklist for Adolescents; DSM, Diagnostic and Statistical Manual of Mental Disorders; GAS-7, 7-item Gaming Addiction Scale; GDS-F, Gaming Disorder Scale—Family; ICD-11, International Classification of Diseases, 11th Revision; IGD, internet gaming disorder; IGDS9-SF, Internet Gaming Disorder Scale–Short-Form; IGDT-10, Ten-Item Internet Gaming Disorder Test; N/A, not applicable; PIE-9, Personal Internet Gaming Disorder Evaluation-9; VGAQ, Videogame Addiction Questionnaire; YDQ, Young Diagnostic Questionnaire.

a Answering “Often” to either Question 9 or 10 of the IGDT-10 counts as one point.

Two meta-analyses on DSM-5-based internet gaming disorder measures found that the Internet Gaming Disorder Scale–Short-Form (IGDS9-SF) was the most widely studied and validated.^19^^,^^70^ The IGDS9-SF includes all 9 DSM-5 criteria for internet gaming disorder, and a systematic review of its psychometrics reported adequate internal consistency and criterion validity.^71^

A priority for future research would be to increase consistency of assessment across studies to enhance the evidence base for select measures. Few measures have been evaluated using clinical samples. Clinical practice incorporates observation and clinical interviews alongside screening tools, and further research is necessary to evaluate diagnostic criteria for disordered gaming and their alignment with clinical practice. While DSM-5 and ICD-11 criteria have been useful, a more robust theoretical framework for disordered gaming may facilitate advancements in assessment and treatment research.^72^

## Epidemiology and prevalence

Across all ages, two meta-analyses found the prevalence of gaming disorder to be 3.05% and 3.3%.^22^^,^^73^ A global meta-analysis of internet gaming disorder in adolescents and young adults between ages 8–28 across 33 countries found an overall prevalence of 9.9%, with rates of 8.8% in adolescents (age 8–18) and 10.4% in young adults (age 18–28).^30^ Another meta-analysis of 16 studies found the prevalence of internet gaming disorder to be 4.6% in adolescents ages 10–19, with higher rates in males (6.8%) compared to females (1.3%).^74^ Smaller samples were associated with higher prevalence of gaming disorder.^74^ Prevalence estimates also varied by geographic region, with some Asian study populations reporting higher prevalence estimates of gaming disorder.^22^^,^^74^ More stringent sampling methods were associated with lower prevalence estimates. When analyses were restricted to studies using more stringent sampling methods, such as representative or random cluster sampling, the overall prevalence of gaming disorder decreased from 3.05% to 1.96%.^22^ Finally, these meta-analyses included studies prior to 2013 when the internet gaming disorder criteria were first published, potentially conflating problematic video game use with internet gaming disorder.

Notably, problematic gaming was typically more common among males than among females.^74^, ^75^, ^76^, ^77^ This may be due, in part, to video game use and longer gaming duration generally being more common in males, as well as greater social acceptability of gaming among males.^24^ Findings further suggest that maladaptive cognitive patterns in male adolescents, such as overvaluing the rewards of playing video games and higher urges to play video games, may explain the apparent sex disparities.^23^

Problematic video game use may also differ by age. As onset is most common during adolescence and young adulthood,^78^ these developmental stages represent critical periods for prevention, detection, and early intervention.^79^ Video game use also typically increases as adolescents get older. The percentage of adolescents using video games for more than 2 h increases from 15.5% at ages 9–11 to 32.1% at ages 11–13.^80^ Adolescence is a period of heightened sensitivity in reward circuitry, and gaming disorder is associated with altered reward processing, which may be associated with increased risk in adolescents for internet gaming disorder and video game addiction.^81^, ^82^, ^83^ Similarly, adolescence is a period of heightened vulnerability to internalizing symptoms and disorders, which are in turn associated with greater risk for gaming disorder.^25^^,^^84^

Regarding racial and ethnic differences, evidence suggests that problematic video game use may be more common among Black, Hispanic, and Native American adolescents compared to their White peers. Notably, past studies have shown that Black, Hispanic, and Asian adolescents have higher video game time overall compared to White peers.^76^^,^^85^^,^^86^ There are also disparities across geographic regions. For instance, East Asian study populations typically have higher rates of gaming disorder, perhaps because gaming is a particularly popular hobby in East Asian cultures.^22^^,^^74^

Disparities in the prevalence of problematic video game use have also been found for sexual and gender minority adolescents. This may partly reflect higher exposure to social exclusion due to their identity, which may be associated with greater digital activity. Video gaming may also serve as a coping mechanism for sexual and gender minority adolescents in response to minority status-related stressors. Sexual and gender minority adolescents have reported using video games to cope with stress and experience positive emotions.^76^^,^^85^^,^^87^, ^88^, ^89^, ^90^, ^91^

Finally, evidence suggests that there are disparities in problematic video game use by household income. Lower household income has been associated with greater overall and problematic video game use, potentially reflecting fewer financial resources to support other recreational activities.^76^ Reduced access to recreational spaces and extracurricular programming may increase reliance on digital activities, and higher levels of video game use in these contexts may increase risk for problematic video game use.

## Risk/maintenance factors and correlates

Key risk factors for problematic video game use and disordered gaming, including demographic, mental health, parenting, and use-pattern factors, are summarized in Fig. 2. Early initiation of gaming is one important risk factor. For instance, adolescents who initiated weekly video game use before age five had a higher risk of problematic video game use compared to those who initiated after age 10.^92^ Preschoolers who initiated video game use were also 1.7 times more likely to be at high risk of internet gaming disorder.^93^ Initiating video game use at a younger age may reflect a stronger tendency for youth to associate gaming with their self-worth, with lower gaming-contingent self-worth proposed to underlie the relationship between earlier initiation and internet gaming disorder.^94^ Moreover, the type of video game use may also be associated with problematic gaming.^95^^,^^96^ For example, in a study of Dutch adolescents and young adults, highly problematic gamers were more likely to spend more time on online versus offline games.^97^

**Fig. 2:**
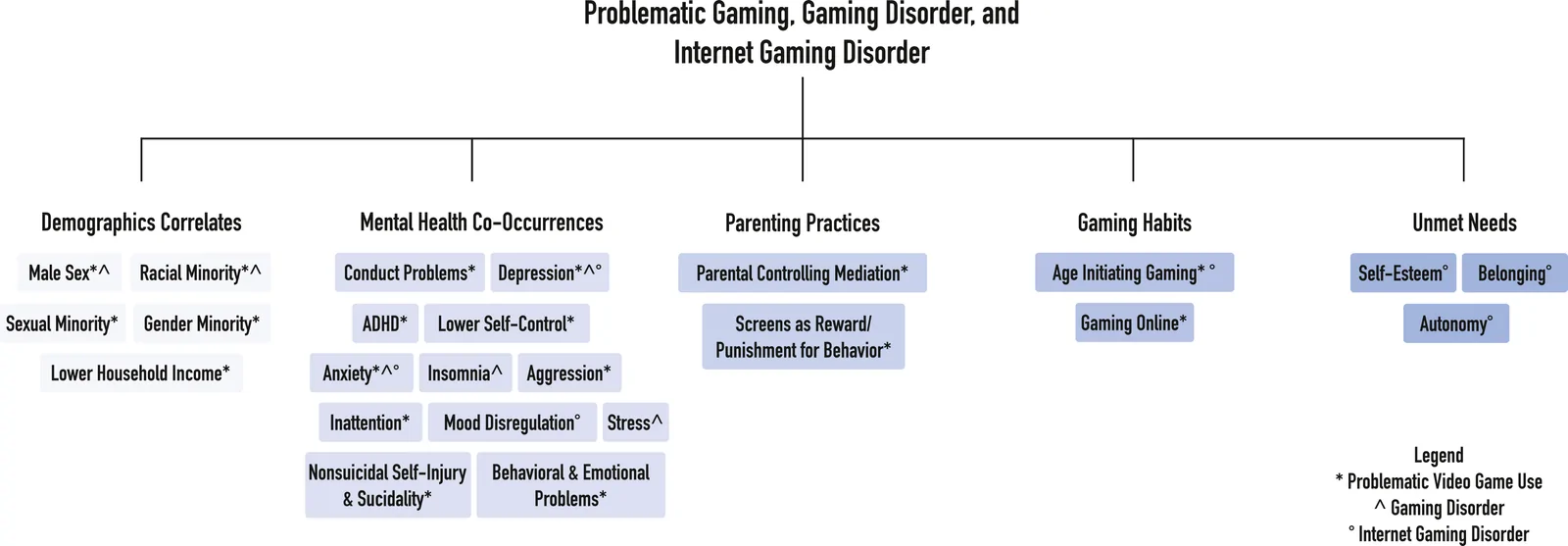
Risk factors for problematic gaming, gaming disorder, and internet gaming disorder. Abbreviation: ADHD, attention-deficit/hyperactivity disorder. Parenting practices and gaming habits are modifiable risk factors.

Certain dispositional traits are also linked to problematic video game use. Previous literature found that more withdrawn behavior, social phobia, and neglect of social contacts were associated with internet gaming disorder, as were lower levels of belonging, self-esteem, and autonomy.^33^^,^^98^ Reciprocal processes may underlie such relationships. For instance, socially withdrawn or anxious individuals may be more likely to turn to video games to avoid traditional social interaction, and those with problematic video game use patterns may end up neglecting social connection and becoming more withdrawn.

Furthermore, parenting practices may also be related to problematic video game use. Along with using screens to reward or punish adolescent behavior, greater parental controlling mediation (i.e., using parental authority and pressure to exert control instead of explaining why things should be a certain way) has been found to be associated with greater problematic gaming behaviors, independent of gaming frequency.^31^ Consequently, controlling parental behaviors may unintentionally encourage adolescents to play video games as a compensatory source of autonomy, thereby potentially increasing the risk of problematic video game use and gaming disorder.^32^^,^^99^, ^100^, ^101^

## Health outcomes

Problematic gaming is associated with a range of adverse physical outcomes. For example, gaming and internet addiction were found to be associated with higher rates of obesity in adolescents, which may be related to unhealthy eating habits (e.g., poorer nutrition, emotional eating) and physical activity displacement.^44^ Gaming disorder has also been associated with a variety of physical symptoms, including joint and muscle pain, headache, vision problems, and sleep disturbances, which was the most frequently reported symptom.^40^ These symptoms were more frequently reported by teenagers.

Sleep is a particularly important area of concern in relation to problematic video game use. A recent systematic review and meta-analysis revealed that problematic gaming is associated with multiple forms of sleep disruption, including shorter sleep duration, poorer sleep quality, higher daytime sleepiness, and increased sleep problems.^39^ Additional sleep disturbances include delayed sleep schedules, sleep deprivation, and nocturnal awakenings. Notably, the effect sizes of these associations were greater than that of other digital media usage, which may reflect the more arousing nature of video games compared to other “passive” media. Problematic gaming was also more strongly associated with sleep duration in adolescents than in adults. This may be related to both late-night gaming behaviors and developmental susceptibility to delayed circadian rhythms during adolescence. Potential mechanisms of sleep disruption include displacement of sleep or other sleep promoting activities, such as exercise, with gaming, thereby reducing overall sleep duration. Blue light exposure from screens can also promote arousal and suppress nighttime melatonin secretion, disrupting normal sleep–wake cycles and increasing sleep latency. Physical symptoms related to gaming such as muscle pain can also disrupt sleep.

Problematic video game use has also been associated with differences in brain development and function. Individuals with gaming disorder^33^^,^^38^^,^^41^^,^^43^^,^^45^ exhibit altered neurobiological structure and function associated with emotion regulation, cognitive control, attention, reward processing, inhibition, self-identity, and several domains of cognitive functioning, comparable to substance addiction.^33^^,^^38^^,^^41^, ^42^, ^43^^,^^45^ Functional neuroimaging reveals that internet gaming disorder is associated with elevated loss sensitivity in brain regions involved with reward circuitry and altered activity in regions responsible for self-monitoring, development of habitual behaviors, and decision-making. Structural brain differences associated with internet gaming disorder include decreased gray-matter volume and white-matter integrity in regions associated with impulse control, emotional regulation, and attention.^33^^,^^38^^,^^41^, ^42^, ^43^^,^^45^ Structural and functional differences have also been found in regions involved in impulse control, identity formation, emotion regulation, and reward sensitivity in problematic gaming.^42^ These findings suggest that an imbalance between weakened cognitive control and a heightened reward system may be associated with both problematic gaming^42^ and internet gaming disorder.^42^^,^^43^^,^^45^

Overall, problematic gaming is associated with poorer physical health outcomes. Problematic gaming is associated with physical symptoms and obesity, which may be related to unhealthy eating habits and displacement of physical activity. Problematic video game use and internet gaming disorder are also associated with poorer sleep quality and lower sleep duration through biological mechanisms and displacement of sleep time. Video game addiction and internet gaming disorder are associated with a variety of structural and functional differences in several brain regions involving reward circuitry and executive functioning.

## Mental health correlates

Problematic gaming is closely intertwined with broader mental health and may act as both a correlate and potential risk factor for psychosocial difficulties. Among adolescents, problematic gaming is commonly associated with attention-deficit/hyperactivity disorder, inattention, anxiety, depression, and behavioral and emotional problems.^49^ Lower self-control has also been linked to problematic gaming, suggesting that impulsivity may contribute to its development.^53^ Relatedly, both problem gambling and problematic gaming are consistently associated with conduct problems and depressive symptoms.^51^ In students and adolescents, problematic gaming has also been associated with increased risk of both nonsuicidal self-injury and suicidality, with anxiety identified as both a mediator and moderator of this relationship.^52^

Evidence from the COVID-19 pandemic further highlights contextual factors that may exacerbate gaming-related harms. During this period, gaming disorder was associated with maladaptive coping regulatory styles, poor social support, higher academic stress, excessive use of social networks, and poorer mental health.^48^ Gaming disorder was also significantly associated with higher levels of depression, anxiety, stress, and insomnia, as well as lower quality of life and subjective well-being.^25^

Importantly, emerging research suggests that these relationships are often bidirectional. Internet gaming disorder, for example, shows reciprocal associations with depression and aggression, indicating that symptoms of depression or aggression may both contribute to and co-occur with disordered gaming.^54^ Internet gaming disorder has also been linked to mood dysregulation and lower psychological well-being, life satisfaction, and academic performance.^50^

## Prevention and treatment

Contemporary evidence supports the application of multimodal, integrative approaches for the treatment of disordered gaming in adolescents. Cognitive behavioral therapy (CBT) remains the most frequently studied form of intervention; however, recent systematic reviews and meta-analyses indicate that CBT is most effective when delivered as part of broader programs incorporating motivational, psychoeducational, and skills-based components (e.g., emotion regulation, self-efficacy), rather than as a standalone treatment.^58^^,^^62^ Across studies, CBT-based and related psychosocial interventions are associated with significant reductions in gaming disorder severity and gaming time, with pooled effect sizes indicating moderate to large improvements.^59^^,^^63^

Importantly, treatment outcomes vary depending on how they are measured. Meta-analytic data suggest consistent reductions in gaming time and disorder symptom severity, as well as moderate improvements in comorbid symptoms such as depression and anxiety.^59^ However, improvements in time spent gaming do not always correspond directly to functional recovery, such as academic performance or social functioning, which are less consistently measured across trials.^58^ This heterogeneity in outcome definitions remains a key limitation in the literature.

Parental involvement is a critical component of treatment in child and adolescent populations. Interventions that integrate parental psychoeducation and communication strategies are commonly included within CBT-based programs and may improve treatment engagement and adherence, even when additional symptom reduction is not consistently observed.^62^^,^^58^ In addition to structured interventions, consistent parental monitoring, structured routines, and early parental recognition of warning signs, such as social isolation, depressive symptoms, and low academic performance, play an important role in prevention.^102^

A range of other non-pharmacological interventions has also been explored. These include behavioral and mindfulness-based interventions, as well as emerging modalities such as virtual reality and animal-assisted therapy, which may support improvements in emotion regulation and interpersonal functioning.^61^ Meta-analytic evidence suggests that non-pharmacological interventions overall are associated with significant reductions in gaming disorder severity, with psychotherapy-based approaches demonstrating the largest effects.^63^ Preventive efforts emphasize school-based and public health approaches, as well as digital well-being programs that promote healthy technology use and early risk identification.^56^

Pharmacological treatments have been investigated primarily in the context of comorbid psychiatric conditions. Evidence from systematic reviews and meta-analyses suggests that several medication classes, including antidepressants (e.g., bupropion, selective serotonin reuptake inhibitors), stimulants for attention-deficit/hyperactivity disorder, and other psychotropic agents, have been trialed, often in combination with psychosocial interventions.^55^^,^^58^^,^^64^ While some studies report improvements in gaming-related outcomes, effects are generally modest and appear to be mediated by improvements in underlying symptoms such as impulsivity, inattention, or depression rather than direct effects on gaming behavior itself. Combined treatment approaches (pharmacotherapy plus psychosocial interventions) may offer enhanced benefit compared to either modality alone in some populations, although further research is needed.^55^

To translate the existing evidence base into actionable clinical practice, we propose a pragmatic clinical pathway (Fig. 3) outlining a stepwise approach to screening, triage, initial management, and referral. This framework synthesizes current knowledge on risk factors, assessment tools, and treatment strategies to inform clinical decision-making despite ongoing limitations in standardization.

**Fig. 3:**
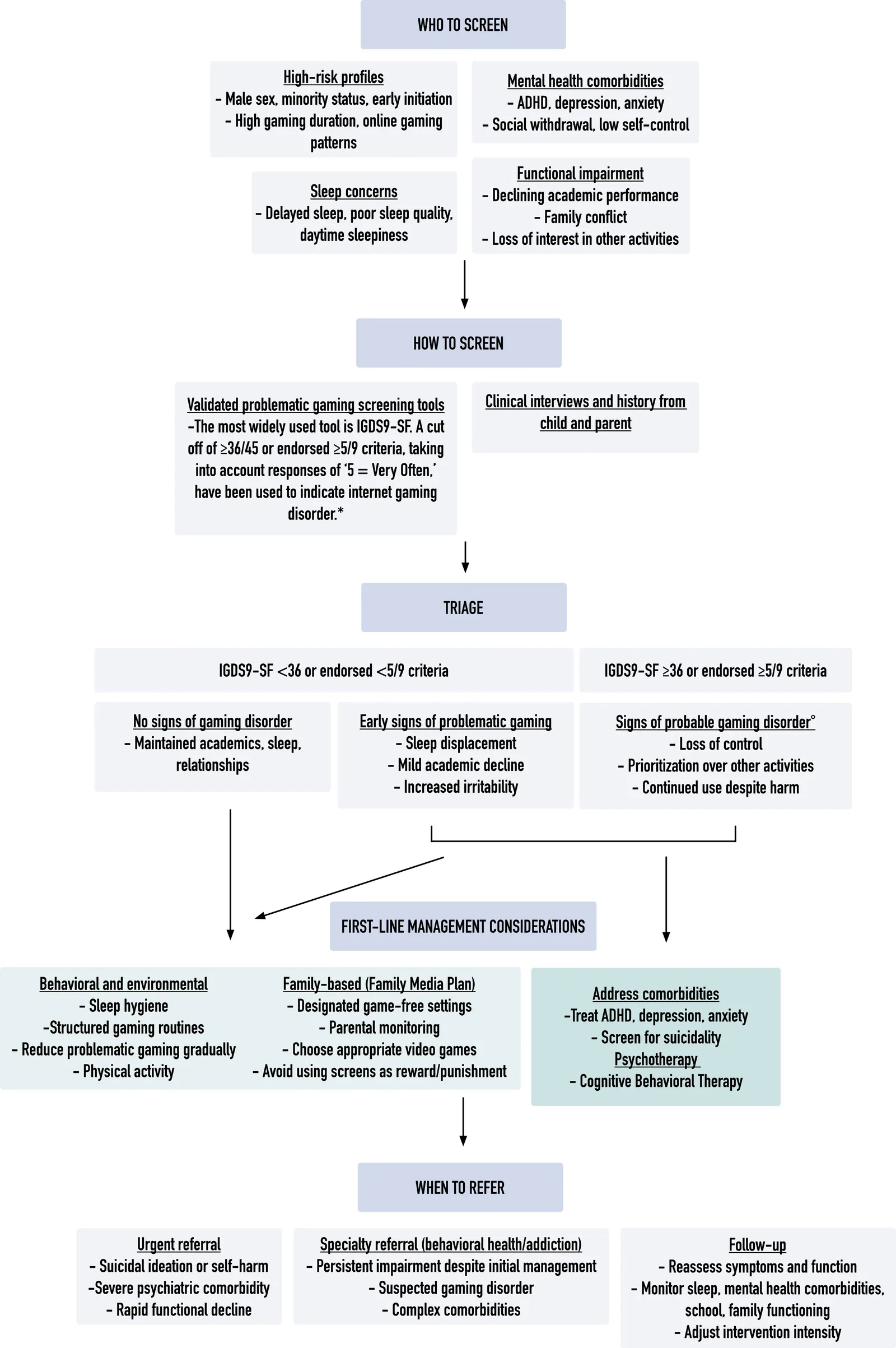
Clinical pathway for screening, triage, and management of problematic gaming, gaming disorder, and internet gaming disorder. Abbreviations: ADHD, attention-deficit/hyperactivity disorder; IGDS9-SF, Internet Gaming Disorder Scale–Short-Form. Suggestions presented in the current figure are based on low-to-moderate narrative certainty, as summarized in Supplemental Table S1. ∗Reported cutoffs vary depending on study population, methodology, and intended use. See Table 3 for details of screening tools. °The signs of probable gaming disorder also encompass the early signs listed previously.

In sum, current evidence supports CBT-based, multimodal interventions with family involvement as first-line treatment, combined with psychosocial or pharmacologic strategies when clinically indicated. However, further high-quality, long-term studies using standardized definitions of both behavioral and functional outcomes are needed to establish clear, evidence-based treatment guidelines for adolescents with disordered gaming.

## Limitations

This review has several limitations. First, heterogeneity in definitions of problematic video game use, gaming disorder, and internet gaming disorder, alongside the lack of standardized assessment and screening measures, diagnostic tools, and outcome metrics for problematic video game use and disordered gaming, hindered cross-study comparisons. As this review synthesized evidence from systematic reviews and meta-analyses with substantial heterogeneity in study designs, populations, and quality assessment approaches (Table 2), we did not assign strength-of-evidence ratings to individual risk factors or health outcomes, as doing so would risk oversimplification. Second, we did not search local-language databases, which precluded coverage of reviews not indexed in PubMed, Web of Science, or PsycInfo. Third, the evidence synthesized by most of the included reviews is cross-sectional, limiting causal inference. Together, these limitations highlight the need for uniform criteria^103^^,^^104^ and longitudinal research to strengthen the evidence base.

## Future directions

To enhance prevention and treatment efforts, future research must address several critical gaps. Consensus committees across disciplines could establish clinical and diagnostic standards,^105^ similar to approaches used in the Red Book, or through consensus-based research methods such as concept mapping.^106^ Such efforts could also inform future iterations of diagnostic manuals, including the next DSM revision, by incorporating standardized criteria for internet gaming disorder/gaming disorder to improve diagnostic consistency. Importantly, these challenges are not limited to gaming; other forms of problematic screen use, such as social media addiction and problematic internet use, face similar issues with definitions, diagnostic criteria, and measurement. Prior work has shown positive associations across multiple behavioral addictions, with problematic internet use, internet gaming disorder, and social media clustering primarily via a shared negative association with conscientiousness,^107^ while another study reported only weak interrelationships between internet gaming disorder and social media addiction despite shared links to mental health symptoms.^107^ Together, these findings suggest both commonalities and distinctions across maladaptive technology use behaviors, supporting the need for transdiagnostic assessment and prevention approaches.

Second, more research should focus on risk factors for disordered gaming. Future studies should consider the role of co-occurring mental health disorders, including depression and anxiety, to better understand their interactions with addictive behaviors.^108^ More research is also needed to identify and strengthen protective psychosocial factors, such as parental monitoring, self-regulation skills, and media literacy, which together may promote resilience and reduce susceptibility to disordered gaming.^109^ Future research should also examine potential gender differences in risk factors, symptom presentation, and intervention outcomes.

Third, well-designed, large-scale randomized controlled trials using standardized outcome measures are needed to evaluate the long-term efficacy of pharmacological and psychosocial treatments. Most studies utilize small samples and have short follow-up periods, often limited to immediate post-treatment or short-term follow-up (e.g., three months).^58^^,^^59^^,^^103^^,^^110^ Additionally, as new technology interventions for disordered gaming have arisen, future studies should study the efficacy of these novel treatments. Technology-based interventions have been introduced to reduce gaming intensity and improve awareness, but their effectiveness remains under-researched and requires evaluation across diverse adolescent and young adult populations.^103^

Fourth, neuroimaging research should employ longitudinal designs and multiple neuroimaging methods (e.g., structural magnetic resonance imaging (MRI) and functional MRI, diffusion tensor imaging) to clarify the direction of associations between problematic video game use and brain function and structure, including underlying mechanisms.^82^^,^^108^ Previous studies have also found that video game addiction predicts and is predicted by alterations in reward circuitry, suggesting a potential bidirectional relationship.^108^ There is a need to elucidate how video game addiction may impact neurodevelopment and how pre-existing neurobiological differences may contribute to risk for problematic video game use.

Finally, at a broader systems level, policy and industry accountability must be addressed. Stronger regulatory frameworks are needed to ensure transparency in the design of games, particularly regarding features that contribute to addictive use.^103^^,^^104^ Collaboration with developers to implement ethical design elements, such as game-time limits, warning prompts, and limits on monetization, represents a promising strategy for reducing harm.

## Contributors

JMN conceptualized and designed the study, drafted the initial manuscript, and critically reviewed and revised the manuscript.

CKH drafted the initial manuscript, and critically reviewed and revised the manuscript.

OHH conceptualized and designed the study, drafted the initial manuscript, and critically reviewed and revised the manuscript.

SN conceptualized and designed the study, drafted the initial manuscript, and critically reviewed and revised the manuscript.

KB drafted the initial manuscript, and critically reviewed and revised the manuscript.

SZ drafted the initial manuscript, and critically reviewed and revised the manuscript.

KH drafted the initial manuscript, and critically reviewed and revised the manuscript.

EJL critically reviewed and revised the manuscript.

MAM critically reviewed and revised the manuscript.

JML critically reviewed and revised the manuscript.

All authors read and approved the final version of the manuscript.

JMN, CKH, SN, KB, SZ, KH accessed and verified the underlying data.

## Data sharing statement

No new participant-level or primary study data were collected or generated for this overview. All data synthesized in this study were derived from previously published systematic reviews and meta-analyses.

## Declaration of interests

OHH and SN received grants from Lookup.Live and #HalfTheStory. The opinions and assertions expressed herein are those of the author(s) and do not reflect the official policy or position of the Uniformed Services University of the Health Sciences or the Department of War.
