# Supplementary material for: Overview of systematic reviews and meta-analyses on problematic video game use and gaming disorder in adolescents and young adults
Source: eClinicalMedicine. 2026 Sep 28;100:104195. doi: 10.1016/j.eclinm.2026.104195 (PMC13639600; doi:10.1016/j.eclinm.2026.104195)
Supplement: Supplementary file 1 [file mmc1.docx]

**Supplementary Materials**

Nagata JM, Helmer CK, Huang OH, Nayak S, Bao K, Zhang S, Handoko K, Li EJ, Moreno MA, Lavender JM. Overview of Systematic Reviews and Meta-Analyses on Problematic Video Game Use and Gaming Disorder in Adolescents and Young Adults. eClinical Medicine. 2026.

|  | **Page** |
| --- | --- |
| **Supplemental Table S1.** Quality appraisal summary and narrative certainty for included systematic reviews and meta-analyses | 2 |
| **Supplemental Table S2.** Coverage of DSM-5 and ICD-11 criteria in selected problematic gaming, internet gaming disorder, and gaming disorder assessment tools | 4 |
| **Supplemental Figure S1.** Study selection flowchart: assessment | 5 |
| **Supplemental Figure S2.** Study selection flowchart: epidemiology and prevalence | 6 |
| **Supplemental Figure S3.** Study selection flowchart: risk/maintenance factors | 7 |
| **Supplemental Figure S4.** Study selection flowchart: health outcomes | 8 |
| **Supplemental Figure S5.** Study selection flowchart: mental health correlates | 9 |
| **Supplemental Figure S6.** Study selection flowchart: prevention and treatment | 10 |

| **Supplemental Table S1.** Quality appraisal summary and narrative certainty for included systematic reviews and meta-analyses | | | | | | |
| --- | --- | --- | --- | --- | --- | --- |
| **Domain** | **Number of reviews** | **Types of included reviews** | **Quality appraisal summary** | **Key limitations** | **Narrative certainty** | **Rationale** |
| Assessment | 3 | 3 systematic reviews | Kmet quality checklist; 2 not assessed | No clinical samples; no gold standard; heterogeneous instruments | Low-Moderate | Only 1/3 reviews used formal appraisal, which would push toward Low. However, findings were highly consistent across all 3 reviews (IGDS9-SF consistently identified as best-supported), which pulls toward Moderate. The balance yields Low-Moderate. |
| Epidemiology and prevalence | 8 | 5 systematic reviews and meta-analyses, 2 systematic reviews, 1 meta-analysis | JBI, AHRQ, NOS, Rosenthal's fail-safe N. Best Bets Critical Appraisal Worksheets, Egger’s; 1 not assessed | High heterogeneity; publication bias (LFK = -3.8); inconsistent instrumentation | Low-Moderate | 7/8 reviews conducted quality appraisal, and prevalence estimates broadly converge (1-6% general population, higher in specific subgroups). However, high heterogeneity across primary studies and evidence of publication bias prevent a Moderate rating. |
| Risk/maintenance factors and correlates | 8 | 1 systematic review and meta-analysis, 2 meta-analyses, 5 systematic reviews | AHRQ, ROBUST, Downs & Black, 10-item checklist, NIH QAT; 3 not assessed | Predominantly cross-sectional primary studies; limited causal inference | Low | Only 5/8 reviews conducted appraisal. Findings are fairly consistent (family dysfunction, depression, poor academics repeatedly identified), but the primary studies are almost entirely cross-sectional, precluding causal inference. This fundamental design limitation keeps the rating at Low. |
| Health outcomes | 11 | 5 systematic reviews, 3 meta-analyses, 2 systematic reviews and meta-analyses, 1 critical review | JBI, NOS, multi-domain RoB, jackknife sensitivity, Egger's, ROBINS-I; 4 not assessed | Small neuroimaging samples; no blinding; limited confound control; heterogeneous outcome measures | Low | 7/11 reviews conducted appraisal, but the domain spans neuroimaging, sleep, and physical health—each with distinct methodological challenges. Neuroimaging studies (which dominate this domain) have small samples, no blinding, and limited confound control. Sleep and BMI data are largely cross-sectional with heterogeneous outcome measures. Despite some consistency in neuroimaging findings (fronto-striatal involvement), the methodological limitations are substantial enough to warrant Low. |
| Mental health correlates | 8 | 5 systematic reviews, 1 systematic review and meta-analysis, 1 meta-analysis, 1 systematic review and meta-analysis | MMAT, NIH QAT, NOS, SANRA, JBI, QATSO, Egger's; 1 not assessed | Predominantly cross-sectional primary studies; directionality unclear | Low-Moderate | 7/8 reviews conducted appraisal, and findings are highly consistent across reviews (depression, anxiety, ADHD, suicidality all repeatedly associated). The primary studies are predominantly cross-sectional (limiting causal inference), but the sheer consistency and volume of evidence across 8 reviews with mostly adequate appraisal supports Low-Moderate. |
| Prevention and treatment | 15 | 11 systematic reviews, 2 systematic reviews and meta-analyses, 2 meta-analyses | Cochrane RoB 2, EPHPP, NOS, JBI, AMSTAR 2, Egger's/Begg's, CONSORT; 5 not assessed | Few high-quality RCTs in primary studies; short follow-up; publication bias | Low-Moderate | 10/15 reviews conducted appraisal, and CBT is consistently supported across reviews. However, primary studies include few high-quality RCTs, short follow-up periods, and evidence of publication bias. The inclusion of some RCTs (unlike other domains) and consistent directionality of findings supports Low-Moderate rather than Low. |
| Narrative certainty ratings were assigned to each domain based on three criteria: (1) the proportion of included reviews that conducted formal quality appraisal of their primary studies, (2) the consistency of findings across reviews within the domain, and (3) the severity of methodological limitations in the underlying primary study designs. Domains were rated as Low-Moderate when findings were consistent across reviews and the majority of reviews conducted formal appraisal, and as Low when fundamental design limitations (e.g., exclusively cross-sectional data, small sample sizes, absence of blinding) substantially constrained the strength of conclusions.  **Abbreviations:** AHRQ, Agency for Healthcare Research and Quality; AMSTAR 2, A MeaSurement Tool to Assess systematic Reviews, version 2; CONSORT, Consolidated Standards of Reporting Trials; Downs & Black, Downs and Black checklist; EPHPP, Effective Public Health Practice Project Quality Assessment Tool; JBI, Joanna Briggs Institute Critical Appraisal Tools; MMAT, Mixed Methods Appraisal Tool; NIH QAT, National Institutes of Health Quality Assessment Tool for Observational Cohort and Cross-Sectional Studies; NOS, Newcastle-Ottawa Scale; QATSO, Quality Assessment Tool for Systematic Reviews of Observational Studies; RoB 2, Cochrane Risk of Bias 2 tool; ROBINS-I , Risk of Bias in Non-randomised Studies; ROBUST, Risk Of Bias in non-randomized Studies of exposures for Use in Systematic reviews of Transversal studies; SANRA, Scale for the Assessment of Narrative Review Articles. | | | | | | |

| **Supplemental Table S2.** Coverage of DSM-5 and ICD-11 criteria in selected problematic gaming, internet gaming disorder, and gaming disorder assessment tools | | | | | | | | | | |
| --- | --- | --- | --- | --- | --- | --- | --- | --- | --- | --- |
|  | Young Diagnostic Questionnaire (YDQ) | Compulsive Internet Use Scale (CIUS-14) | 7-item Gaming Addiction Scale (GAS-7) | Internet Gaming Disorder Scale—Short Form (IGDS9-SF) | 9-item dichotomous Internet Gaming Disorder Scale (IGD-9) | Personal Internet Gaming Disorder Evaluation-9 (PIE-9) | Ten-Item Internet Gaming Disorder Test (IGDT-10) | Gaming Disorder Scale—Family (GDS-F) | Development of the DSM–5 IGD Symptoms Checklist for Adolescents (DISCA) | Videogame Addiction Questionnaire (VGAQ) |
| **DSM-5 Criteria for Internet Gaming Disorder**^a^ |  |  |  |  |  |  |  |  |  |  |
| Preoccupation with Internet games. | X | X | X | X | X | X | X | X | X | X |
| Withdrawal symptoms when Internet gaming is taken away | X | X | X | X | X | X | X | X | X | X |
| Tolerance—the need to spend increasing amounts of time engaged in Internet games. | X |  | X | X | X | X | X | X | X | X |
| Unsuccessful attempts to control the participation in Internet games. | X | X | X | X | X | X | X | X | X | X |
| Loss of interests in previous hobbies and entertainment as a result of, and with the exception of, Internet games. |  |  | X | X | X | X | X | X | X |  |
| Continued excessive use of Internet games despite knowledge of psychosocial problems. |  |  |  |  |  | X | X | X | X |  |
| Has deceived family members, therapists, or others regarding the amount of Internet gaming. | X | X |  | X | X | X | X | X | X |  |
| Use of Internet games to escape or relieve a negative mood | X | X | X | X | X | X | X | X | X | X |
| Has jeopardized or lost a significant relationship, job, or educational or career opportunity because of participation in Internet games. | X | X | X | X | X | X | X | X | X | X |
| **ICD-11 Criteria for Gaming Disorder** |  |  |  |  |  |  |  |  |  |  |
| Impaired control over gaming | X | X | X | X | X | X | X | X | X | X |
| Increasing priority given to gaming to the extent that gaming takes precedence over other life interests and daily activities |  | X | X | X | X | X | X | X | X |  |
| Continuation or escalation of gaming despite the occurrence of negative consequences. |  |  |  | X |  | X | X | X | X |  |
| The behaviour pattern is of sufficient severity to result in significant impairment in personal, family, social, educational, occupational or other important areas of functioning. | X | X | X | X |  | X | X | X | X | X |
| Abbreviations: CIUS-14, Compulsive Internet Use Scale; DISCA, DSM-5 Internet Gaming Disorder Symptoms Checklist for Adolescents; DSM-5, Diagnostic and Statistical Manual of Mental Disorders, Fifth Edition; GAS-7, 7-item Gaming Addiction Scale; GDS-F, Gaming Disorder Scale—Family; ICD-11, International Classification of Diseases, 11th Revision; IGD, internet gaming disorder; IGDS9-SF, Internet Gaming Disorder Scale—Short Form; IGDT-10, Ten-Item Internet Gaming Disorder Test; PIE-9, Personal Internet Gaming Disorder Evaluation-9; VGAQ, Videogame Addiction Questionnaire; YDQ, Young Diagnostic Questionnaire.  ^a^ A diagnosis of internet gaming disorder would require experiencing five or more of these symptoms within a year. The condition can include gaming on the internet with others or alone. | | | | | | | | | | |

**Supplemental Figure S1.** Study selection flowchart: assessment

PubMed and Web of Science Search: (“problematic video game use”[tw] OR “problematic gaming”[tw] OR “internet gaming disorder”[tw] OR “gaming disorder”[tw] OR “excessive gaming”[tw] OR “video game addiction”[tw]) AND (assessment[tw] OR screening[tw] OR measurement[tw] OR evaluation[tw] OR scale[tw] OR questionnaire[tw]) AND (adolescen*[tw] OR youth[tw] OR teen*[tw] OR “young adult*”[tw])

PsycInfo Search: (“problematic video game use” OR “problematic gaming” OR “internet gaming disorder” OR “gaming disorder” OR “excessive gaming” OR “video game addiction”) AND (assessment OR screening OR measurement OR evaluation OR scale OR questionnaire) AND (adolescen* OR youth OR teen* OR “young adult*”)

Filters applied: meta-analysis, systematic review

Articles identified through databases

(PubMed: n=20, Web of Science: n=11, PsycInfo: n=28)

Records screened

(n=59)

Records excluded (n=11)

Full-text articles reviewed for potential retrieval and eligibility (n=48)

Articles excluded due to irrelevance to assessment (n=45)

Studies included in review (n=3)

**Supplemental Figure S2.** Study selection flowchart: epidemiology and prevalence

PubMed and Web of Science Search: (“problematic video game use”[tw] OR “problematic gaming”[tw] OR “internet gaming disorder”[tw] OR “gaming disorder”[tw] OR “excessive gaming”[tw] OR “video game addiction”[tw]) AND (epidemiology[tw] OR prevalence[tw] OR incidence[tw]) AND (sociodemographic*[tw] OR demographic*[tw] OR sex[tw] OR gender[tw] OR age[tw] OR race[tw] OR ethnic*[tw] OR income[tw] OR “socioeconomic status”[tw] OR neighborhood[tw]) AND (adolescen*[tw] OR youth[tw] OR teen*[tw] OR “young adult*”[tw])

PsycInfo Search: (“problematic video game use” OR “problematic gaming” OR “internet gaming disorder” OR “gaming disorder” OR “excessive gaming” OR “video game addiction”) AND (epidemiology OR prevalence OR incidence) AND (sociodemographic* OR demographic* OR sex OR gender OR age OR race OR ethnic* OR income OR “socioeconomic status” OR neighborhood) AND (adolescen* OR youth OR teen* OR “young adult*”)

Filters applied: meta-analysis, systematic review

Articles identified through databases

(PubMed: n=11, Web of Science: n=14, PsycInfo: n=10)

Records screened

(n=35)

Records excluded (n=5)

Full-text articles reviewed for potential retrieval and eligibility (n=30)

Articles excluded due to irrelevance to epidemiology and prevalence (n=22)

Studies included in review (n=8)

**Supplemental Figure S3.** Study selection flowchart: risk/maintenance factors

PubMed and Web of Science Search: (“problematic video game use”[tw] OR “problematic gaming”[tw] OR “internet gaming disorder”[tw] OR “gaming disorder”[tw] OR “excessive gaming”[tw] OR “video game addiction”[tw]) AND (“risk factor*”[tw] OR predictor*[tw] OR correlate*[tw] OR disposition[tw] OR associat*[tw] OR vulnerability[tw]) AND (parenting[tw] OR peer*[tw] OR bullying[tw] OR stress[tw] OR psychosocial[tw] OR famil*[tw]) AND (adolescen*[tw] OR youth[tw] OR teen*[tw] OR “young adult*”[tw])

PsycInfo Search: (“problematic video game use” OR “problematic gaming” OR “internet gaming disorder” OR “gaming disorder” OR “excessive gaming” OR “video game addiction”) AND (“risk factor*” OR predictor* OR correlate* OR disposition OR associat* OR vulnerability) AND (parenting OR peer* OR bullying OR stress OR psychosocial OR famil*) AND (adolescen* OR youth OR teen* OR “young adult*”)

Filters applied: meta-analysis, systematic review

Articles identified through databases

(PubMed: n=9, Web of Science: n=6, PsycInfo: n=9)

Records screened

(n=24)

Records excluded (n=6)

Full-text articles reviewed for potential retrieval and eligibility (n=18)

Articles excluded due to irrelevance to risk/maintenance factors (n=10)

Studies included in review (n=8)

**Supplemental Figure S4.** Study selection flowchart: health outcomes

PubMed and Web of Science Search: ("problematic video game use"[tw] OR "problematic gaming"[tw] OR "internet gaming disorder"[tw] OR "gaming disorder"[tw] OR "excessive gaming"[tw] OR "video game addiction"[tw]) AND ("health outcome*"[tw] OR obes*[tw] OR BMI[tw] OR "physical health"[tw] OR sleep*[tw] OR "circadian rhythm"[tw] OR cardiovascular*[tw] OR "functional neuroimaging"[tw] OR "structural neuroimaging"[tw] OR neurobiolog*[tw] OR "brain development"[tw] OR neural) AND (development[tw] OR impairment[tw] OR associat*[tw] OR effect*[tw] OR impact*[tw]) AND (adolescen*[tw] OR youth[tw] OR teen*[tw] OR "young adult*"[tw])

PsycInfo Search: (“problematic video game use” OR “problematic gaming” OR “internet gaming disorder” OR “gaming disorder” OR “excessive gaming” OR “video game addiction”) AND (“health outcome*” OR obes* OR BMI OR “physical health” OR sleep* OR “circadian rhythm” OR cardiovascular* OR “functional neuroimaging” OR “structural neuroimaging” OR neurobiolog* OR “brain development” OR neural) AND (development OR impairment OR associat* OR effect* OR impact*) AND (adolescen* OR youth OR teen* OR “young adult*”)

Filters applied: meta-analysis, systematic review

Articles identified through databases

(PubMed: n=14, Web of Science: n=0, PsycInfo: n=8)

Records screened

(n=22)

Records excluded (n=6)

Full-text articles reviewed for potential retrieval and eligibility (n=16)

Articles excluded due to irrelevance to health outcomes (n=5)

Studies included in review (n=11)

**Supplemental Figure S5.** Study selection flowchart: mental health correlates

PubMed and Web of Science Search: ("problematic video game use"[tw] OR "problematic gaming"[tw] OR "internet gaming disorder"[tw] OR "gaming disorder"[tw] OR "excessive gaming"[tw] OR "video game addiction"[tw]) AND ("health outcome*"[tw] OR "mental health"[tw] OR depress*[tw] OR anxi*[tw] OR ADHD[tw] OR suicid*[tw] OR "self-harm"[tw] OR aggress*[tw] OR "self-control"[tw]) AND (development[tw] OR impairment[tw] OR associat*[tw] OR effect*[tw] OR impact*[tw] OR relat*[tw]) AND (adolescen*[tw] OR youth[tw] OR teen*[tw] OR "young adult*"[tw])

PsycInfo Search: ("problematic video game use" OR "problematic gaming" OR "internet gaming disorder" OR "gaming disorder" OR "excessive gaming" OR "video game addiction") AND ("health outcome*" OR "mental health" OR depress* OR anxi* OR ADHD OR suicid* OR "self-harm" OR aggress* OR "self-control") AND (development OR impairment OR associat* OR effect* OR impact* OR relat*) AND (adolescen* OR youth OR teen* OR "young adult*")

Filters applied: meta-analysis, systematic review

Articles identified through databases

(PubMed: n=24, Web of Science: n=8, PsycInfo: n=26)

Records screened

(n=58)

Records excluded (n=9)

Full-text articles reviewed for potential retrieval and eligibility (n=49)

Articles excluded due to irrelevance to mental health (n=41)

Studies included in review (n=8)

**Supplemental Figure S6.** Study selection flowchart: prevention and treatment

PubMed and Web of Science Search: (“problematic video game use”[tw] OR “problematic gaming”[tw] OR “internet gaming disorder”[tw] OR “gaming disorder”[tw] OR “excessive gaming”[tw] OR “video game addiction”[tw]) AND (treatment[tw] OR intervention[tw] OR “cognitive behavioral therapy”[tw] OR therap*[tw] OR prevention OR psychoeducation[tw] OR pharmacological[tw]) AND (adolescen*[tw] OR youth[tw] OR teen*[tw] OR “young adult*”[tw])

PsycInfo Search: (“problematic video game use” OR “problematic gaming” OR “internet gaming disorder” OR “gaming disorder” OR “excessive gaming” OR “video game addiction”) AND (treatment OR intervention OR “cognitive behavioral therapy” OR therap* OR prevention OR psychoeducation OR pharmacological) AND (adolescen* OR youth OR teen* OR “young adult*”)

Filters applied: meta-analysis, systematic review

Articles identified through databases

(PubMed: n=28, Web of Science: n=6, PsycInfo: n=28)

Records screened

(n=62)

Records excluded (n=5)

Full-text articles reviewed for potential retrieval and eligibility (n=57)

Articles excluded due to irrelevance to prevention and treatment (n=42)

Studies included in review (n=15)
